# Antibacterial, Antifungal and Antiviral Polymeric Food Packaging in Post-COVID-19 Era

**DOI:** 10.3390/polym14194042

**Published:** 2022-09-27

**Authors:** Atcharawan Srisa, Khwanchat Promhuad, Horman San, Yeyen Laorenza, Phanwipa Wongphan, Kiattichai Wadaugsorn, Janenutch Sodsai, Thitiporn Kaewpetch, Kittichai Tansin, Nathdanai Harnkarnsujarit

**Affiliations:** 1 Department of Packaging and Materials Technology, Faculty of Agro-Industry, Kasetsart University, 50 Ngam Wong Wan Rd., Latyao, Chatuchak, Bangkok 10900, Thailand; 2 Center for Advanced Studies for Agriculture and Food, Kasetsart University, 50 Ngam Wong Wan Rd., Latyao, Chatuchak, Bangkok 10900, Thailand

**Keywords:** antimicrobial, mold, bacteria, virus, active packaging, advanced material, food packaging

## Abstract

Consumers are now more concerned about food safety and hygiene following the COVID-19 pandemic. Antimicrobial packaging has attracted increased interest by reducing contamination of food surfaces to deliver quality and safe food while maintaining shelf life. Active packaging materials to reduce contamination or inhibit viral activity in packaged foods and on packaging surfaces are mostly prepared using solvent casting, but very few materials demonstrate antiviral activity on foods of animal origin, which are important in the human diet. Incorporation of silver nanoparticles, essential oils and natural plant extracts as antimicrobial agents in/on polymeric matrices provides improved antifungal, antibacterial and antiviral properties. This paper reviews recent developments in antifungal, antibacterial and antiviral packaging incorporating natural or synthetic compounds using preparation methods including extrusion, solvent casting and surface modification treatment for surface coating and their applications in several foods (i.e., bakery products, fruits and vegetables, meat and meat products, fish and seafood and milk and dairy foods). Findings showed that antimicrobial material as films, coated films, coating and pouches exhibited efficient antimicrobial activity in vitro but lower activity in real food systems. Antimicrobial activity depends on (i) polar or non-polar food components, (ii) interactions between antimicrobial compounds and the polymer materials and (iii) interactions between environmental conditions and active films (i.e., relative humidity, oxygen and water vapor permeability and temperature) that impact the migration or diffusion of active compounds in foods. Knowledge gained from the plethora of existing studies on antimicrobial polymers can be effectively utilized to develop multifunctional antimicrobial materials that can protect food products and packaging surfaces from SARS-CoV-2 contamination.

## 1. Introduction

According to the latest global estimates from the World Health Organization (WHO), contaminated food results in 600 million cases of foodborne diseases and 420,000 deaths worldwide every year [1,2,3]. Foods are highly susceptible to spoilage, cross-contamination or re-contamination by microorganisms (e.g., bacteria, yeast, molds and viruses) throughout the food chain during food processing, food retail (supermarkets, convenience stores or restaurants) or home storage. Several factors affect quality changes in foods including humidity, oxygen, food chemical components and matrix structures [4,5]. Some microbial strains produce toxins that are harmful to consumers. Spoilage from yeast and mold leads to major economic losses in the food industry and is also detrimental to consumer health. Pathogenic bacteria responsible for food spoilage, poisoning and toxicity such as *Campylobacter* spp., *Clostridium perfringens*, *Escherichia coli*, *Clostridium botulinum*, *Salmonella* spp. and *Listeria monocytogenes* are major hazards to consumer safety [6]. Enteric foodborne viruses such as human norovirus (NoV) and hepatitis A virus (HAV) are of great concern for food safety because they cause gastroenteritis and foodborne illnesses in humans [7,8]. Raw or uncooked foods, fresh produce and ready-to-eat (RTE) foods can easily become cross-contaminated or re-contaminated with foodborne pathogens through contact with food handlers and contact surfaces during processing or packaging. The COVID-19 pandemic is still causing major public health problems in both developed and developing countries. Consumers are increasingly concerned about the possible transmission of SARS-CoV-2 via food and packaging surfaces and its potential effect on food safety. Consumers are also concerned about the safety of synthetic preservatives and their potential health risks. Various technologies have been employed to inactivate or reduce the number of bacteria and viruses including the use of sanitizers or disinfectants, traditional thermal processing technologies and nonthermal processing technologies; however, complete foodborne bacterial removal and viral inactivation of food products are difficult.

**Figure 1:**
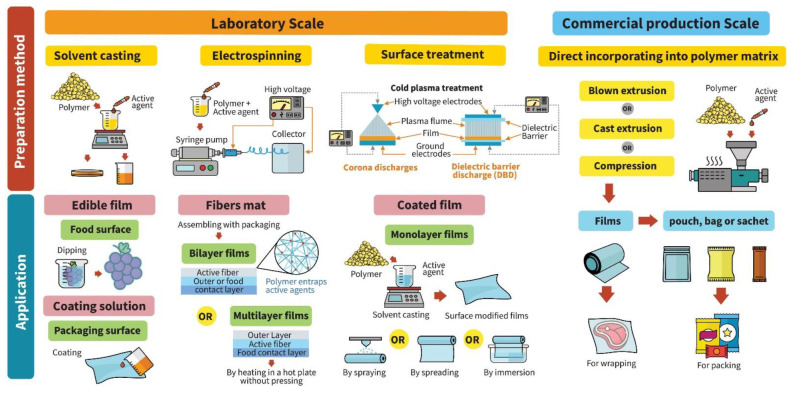
Different incorporation techniques used for developing antimicrobial packaging and their applications on food products.

Functional packaging material with antifungal, antibacterial or antiviral properties is one alternative to preservative addition in bulk foods. Figure 1 presents different incorporation techniques used for developing antimicrobial activity and their applications on food products. Antifungal and antibacterial packaging developed by embedding or coating antibacterial agents in or on packaging materials extends the lag phase and reduces or inhibits the maximum cell count in the stationary phase of yeast, mold and bacterial growth [9,10,11]. Recent research has investigated the antimicrobial efficacy of metal nanoparticles, organic acids and their salts, essential oils, natural extracts, enzymes and bacteriocins incorporated into polymer matrices via different methods including solvent casting, coating solution, surface modification, blown extrusion and cast extrusion. Polymer materials serve as vehicles for loading antimicrobial agents in food packaging such as films, coated films, edible films or coating, pouches and sachets. According to a report by ‘Credence Research’, the market for biodegradable food packaging is expected to reach USD 7058.8 million by the end of 2023, with a compound annual growth rate (CAGR) of 11% from years 2016 to 2023 [12]. Antiviral packaging is designed to improve food safety by either preventing cross-contamination on food surfaces or inactivating target-specific foodborne viruses. Antifungal and antibacterial packaging materials have been intensively studied, but scant research has addressed food-grade polymers and biopolymers with antiviral activity for food applications.

This review of antimicrobial food packaging includes literature covering the previous five years with focus on (i) recent preparation methods of packaging material and recent progress in packaging applications including compression molding, blown extrusion, cast extrusion and solvent casting with either direct addition of antimicrobial agents into the polymer matrix or coating onto the material surface to produce wrapping films, pouches and coating, (ii) antimicrobial efficiency in vitro and in vivo and the challenge test for evaluating the efficiency of antimicrobial material to inhibit the growth of certain microorganisms inoculated intentionally on food products for a certain period and storage condition, (iii) antimicrobial mechanisms against specific microbial food spoilage and foodborne pathogens including yeast and mold, bacteria and viruses by either direct or indirect contact assays that may provide alternative strategies to improve antimicrobial packaging material and (iv) antiviral activity of biobased materials functionalized with natural plant extracts and silver nanoparticles in common viruses such as murine norovirus (MNV) and hepatitis A virus (HAV), with specific focus on surfaces or packaging materials for food products. Future challenges, opportunities and perspectives of using existing knowledge on antimicrobial packaging are discussed to develop multifunctional antimicrobial materials to preserve food safety and quality from spoilage by pathogenic microorganisms and protect food products and packaging surfaces from SARS-CoV-2 contamination.

## 2. Antifungal Food Packaging

### 2.1. Fungal Spoilage in Food Products

Yeast spoilage occurs on or in foods rich in fermentable carbohydrates, high sugar or salt content, low water activity (a_w_), low pH and presence or absence of oxygen [13,14]. Yeasts are found in and cause spoilage of dairy products (cheese and fermented milk), alcoholic beverages (wine and beer), non-alcoholic beverages (soft drinks and fruit juices), fruits, dried and salted meats and fish [15,16]. Spoilage yeasts commonly found growing in food and beverages include species of *Candida*, *Debaryomyces*, *Saccharomyces* and *Zygosaccharomyces* [17,18].

Mold spoilage is favored in food products with high aw, low pH and exposure to headspace oxygen when stored at room temperature or below [19,20]. A wide range of food products including cereals, bakery items, fresh pasta, dairy (cheese and yogurt), fresh fruits and vegetables and dried or salted meat and fish are susceptible to fungal spoilage [21,22]. The main mold species that often cause food spoilage include *Aspergillus* spp., *Fusarium* spp., *Penicillium* spp. and *Rhizopus* spp. [23]. Molds negatively impact the economy of the food industry and are also a health risk due to mycotoxin production by fungal pathogens.

### 2.2. Applications of Active Packaging to Control Fungal Spoilage

Active packaging with anti-yeast or anti-mold activities is used as an alternative strategy to inhibit or retard surface fungal growth as well as maintain food quality (e.g., coating, film and sachet) [24,25]. Antifungal packaging is a feasible option to reduce fungal growth and mycotoxin production. Incorporation of antifungal compounds into the packaging can kill or extend the lag phase or decrease mycelial growth and spore germination during the stationary phase of mold growth, as well as inhibit and/or kill cells in the stationary phase of yeast growth [26,27]. Spoilage inhibition can be performed either by direct contact between packaging material and foods or by indirect contact, whereby the antimicrobial packaging releases a volatile agent into the headspace of the packaging [28]. Recent studies have examined the inhibition of yeast and mold spoilage by direct contact, which is a simple modification of the disk diffusion assay (in vitro test) and can be related to direct contact of the films on food surfaces in the in vivo test.

#### 2.2.1. Effect on Yeast

Yeast species from the genera *Candida* and *Rhodotorula* commonly cause spoilage as foamy, bottle swelling and yeasty off-flavors in yogurt and dairy products [29,30]. Natamycin is mostly incorporated as a polymer on the contact surface of dairy products. Due to its low solubility, it inhibits yeast growth by binding specifically to ergosterol in the yeast cells without permeabilizing the yeast plasma membrane [24,31,32]. Natamycin immobilized on the surface of LDPE film treated for 6 min with UV irradiation demonstrated maximum grafting efficiency (81.96%) and anti-yeast activity against *Rhodotorula mucilaginosa* and *Candida parapsilosis* in the disk diffusion test with diameters of 10.62 and 8.06 mm, respectively, and also decreased yeast population in contaminated Iranian yogurt drink (Doogh) from day 0 to day 23 by 52.33% compared to the control sample (increased by 37.86%) [15]. *Candida albicans* is the most common human fungal pathogen, with causes ranging from mucous membranes to systemic infections [33]. *Candida albicans* showed inhibition in the agar spot diffusion test in the presence of carboxymethyl cellulose coating solution containing 0.05 and 0.5% w/v natamycin, but inhibition was weaker than *Aspergillus flavus* [24]. *Candida* is more resistant to natamycin than mold spores. By contrast, Brzezinska et al. (2019) [27] observed that PLA, PCL or PHB films incorporated with 1.0% polyhexamethyleneguanidine (PHMG) derivatives had a stronger biocidal effect on cells of *Candida albicans* than *Aspergillus niger* and *Penicillium chrysogenum* due to the inhibited activity of *Candida albicans* hydrolases (40 to 52% depending on the type of films). Therefore, natamycin or PHMG derivatives introduced into the polymer can be used as a food contact surface material to reduce the growth of spoilage yeasts in dairy products (e.g., fermented milk and cheese). However, the more resistance or sensitivity of yeast cells to each active material compared with mold spores is unclear.

#### 2.2.2. Effect on Rhizopus, Penicillium and Aspergillus Species

*Aspergillus*, *Rhizopus* and *Penicillium* species are most frequently used as models to study food spoilage fungi by direct contact. Table 1 presents a review of current antifungal packaging based on synthetic and natural antifungal agents incorporated with non-biodegradable and biodegradable materials and their applications in food products.

Fasihnia et al. (2018) [34] found PP films incorporated with 2 to 6% (w/w) sorbic acid were effective against *Aspergillus niger* with inhibition zones of 3.20 and 6.70 cm^2^ after 96 h of incubation, respectively. Da Rocha et al. (2018) [35] observed that Argentine anchovy protein films incorporated with 1.50% (w/v) of sorbic acid or benzoic acid had no effect on *Rhizopus oryzae* after 48 h of incubation. The antimicrobial action of sorbic acid is not well understood but is considered to be based on cytoplasmic acidification and inhibition of the basic metabolic response of the cells [36]. In theory, the antimicrobial effect increases if lipophilic compounds enter the cell more easily [37]. Wangprasertkul et al. (2021) [38] found that PBAT/TPS blend films containing sodium benzoate and/or potassium sorbate gave different antimicrobial performances between in vitro and fresh rice noodles. The presence of oil on the noodle surface accelerated diffusion and enhanced the release of sodium benzoate and potassium sorbate compared to aqueous matrices of agar media and reduced oxygen permeability of the films, resulting in reduced mold growth. Similarly, Küçük et al. (2020) [39] observed that maximum inhibition zone diameters were obtained on zein films with 4000 ppm natamycin at 2.20 and 3.37 cm, while alginate films with 4000 ppm natamycin gave 4.40 and 4.97 cm for *Aspergillus niger* and *Penicillium camamberti*, respectively, but alginate films with natamycin were ineffective against *Penicillium camamberti* in kashar cheese during storage under refrigerated temperature for 45 days compared to in vitro agar medium. Natamycin has low solubility in water with low food penetration but remains on the food surface. Thus, antifungal activity depends on release rate or concentration and composition of food products as well as permeability of the films. Interaction between the polymer and active compounds, as well as miscibility in the polymeric film matrix structure, also reduces water sensitivity, hydrophilicity and solubility that relate to the release of active compounds [40,41,42].

Biological controls using antagonistic yeasts such as *Williopsis saturnus* var. *saturnus* can inhibit spore germination of filamentous fungi, damage the cell membrane and alter protein expression. In whey protein concentrate edible film containing 7 and 9 log CFU/cm^2^, *W. saturnus* gave more efficient inhibition of *Penicillium expansum* and *Aspergillus niger* by 29% and 19%, respectively, after incubation for 5 days on nutritious medium acidified to pH 4.5 and 5.2.

Quantum fillers have recently been introduced as new functional fillers for food packaging biopolymer films. They provide excellent antifungal activity by partially damaging the cell wall structure, penetration and degradation of cellular components [43]. Alginate films with 3 wt% sulfur quantum dot films exhibited strong fungicidal activity against *Aspergillus niger* and *Penicillium chrysogenum* strains by reducing the number and density of conidia and hyphae, with inhibition regions of 14 mm and 18 mm, respectively, after incubation for 3 days, and showed promise by inhibiting mold growth of bread wrapped for 14 days [44].

Volatile active substances (such as herbs, spices, essential oils and their constituents) can be applied either in the direct contact phase or in the vapor phase. A direct contact assay was used to examine the antifungal effectiveness against fungal spoilage on single-packed bakery products (such as buns, white bread or butter cakes) using cellulose acetate films containing 1.5%, 2.5% and 3.5% oregano essential oil. Hamburger buns showed delayed growth of filamentous fungi until the 29th day [45]. PLA/PBSA blend films containing 3 and 6 wt% thymol were able to delay the visible growth of yeast and mold in packed bread by 7 and 9 days, respectively [46]. P(3HB-co-4HB) film with 30% thyme oil extended shelf life of bread to more than 5 days [47]. PBAT/PLA films containing 2 and 5% carvacrol prevented mold growth and extended shelf life of bread and butter cake by 2–4 days [26]. PLA or PBAT blend films containing *trans*-cinnamaldehyde (2%, 5% and 10%) effectively inhibited the microbial growth of bacteria and fungi for more than 21 days [9], while PE film coated with zein containing 0.5% garlic extract and bread aroma maintained bread free of mold infection for 30 days [48]. The extensive concentration of essential oil flavor and scent in packaging materials had a negative effect on the food acceptability in terms of taste and odor.

By contrast, vapor contact offers an alternative way to reduce the development of undesirable flavors. During storage, essential oils and their constituents are released as vapor from the film matrix into the packaging headspace, and this inhibits or delays fungal growth and sporulation. In an in vivo test, a sachet containing a combination of eugenol and citral essential oils in microcapsules showed stronger inhibitory effects on *Penicillium roqueforti* than *Aspergillus niger* in vitro and effectively inhibited colony spots on sliced bread without affecting bread smell or taste (in vapor phase) [25]. Antifungal activity of films has been investigated in vitro by disk diffusion and vapor phase methods. Growth inhibition of *Aspergillus* spp. (61.7–80.45% reduction) and *Penicillium* spp. (55.9–74.1% reduction) in the vapor phase was observed for PLA/PBSA blend film containing 6 wt% thymol, while PLA/PBSA blend film containing 3 wt% thymol showed lower growth reduction of the fungi (15.15–23.16% and 11.49–26.21%, respectively) [46]. Srisa and Harnkarnsujarit (2020) [9] confirmed that essential oils were more effective antifungals on culture media than vapor phases for PLA or PBAT blend films containing *trans*-cinnamaldehyde. Klinmalai et al. (2021) [26] stated that minimal affinity between agar media and film components limited direct diffusion of essential oils into agar media, while the main antifungal mechanism occurred via release of volatile compounds into the headspace, followed by absorption into media matrices.

#### 2.2.3. Effect on Phytopathogenic Fungus

*Colletotrichum gloeosporioides* and *Botrytis cinerea* are fungi responsible for the plant disease anthracnose that causes harvest losses. LDPE films with 3.5% w/w lauric acid affected growth of *Colletotrichum tamarilloi* by decreasing mycelial size and also impacted the cottony characteristic of the fungus in vitro. The presence of disease symptoms, as growth of mycelia in the peduncle or skin breakage in tree tomato, was not observed for about two weeks for the yellow variety and one week for the red variety stored at 27 °C [10]. Biodegradable films containing carbon quantum dots [43] and coffee parchment waste extracts [49] exhibited high antifungal activity against *Colletotrichum tamarilloi* in the in vitro assay. In gaseous contact, PLA films incorporated with 15% R-(−)-carvone and 20% thymol were the most effective at 12 °C in suppressing mycelial growth of avocado and citrus *Colletotrichum gloeosporioides* isolates, respectively, whereas film incorporated with 20% thymol had the highest antifungal activity against both anthracnose isolates at 25 °C [50]. In another case, quinoa protein/chitosan coating with 10% dilution of thymol nano-emulsion showed a significant decrease in yeast and mold of 4.7 log CFU/cm^2^ in artificially inoculated *Botrytis cinerea* cherry tomatoes after 7 days at 5 °C [51], and polyvinyl alcohol/corn starch blends decreased the disease incidence level by up to 27% and by around 40% of infected fruit for *Botrytis cinerea* and *Penicillium expansum* for 12 days, respectively. Damage severity was reduced by around 30% and by 33% with respect to the control samples when using carvacrol-loaded coatings for *Botrytis cinerea* and *Penicillium expansum*, respectively. Severe damage to the fungal membranes and cell walls led to morphological deformations, collapse and deterioration of the conidia and/or hyphae [52].

*Trichoderma* sp. is a mushroom pathogen causing green mold on the commercially cultivated white button mushroom *Agaricus bisporus*. *Trichoderma* sp. isolated from postharvest white mushrooms can be inhibited by sodium alginate films with *β*-cyclodextrin microencapsulated carvacrol [53] and corn starch/polyvinyl alcohol blend films with carvacrol nano-emulsions [54]. The authors indicated that antifungal activity of the polysaccharide-based films against *Trichoderma* sp. might be due to inhibition of spore germination and a chemical effect or physical destruction during the action of carvacrol release when the films were directly in contact with agar media.

#### 2.2.4. Effect on Aflatoxigenic Fungi

The most common fungal species associated with aflatoxin production of cereals are *Aspergillus flavus* and *Aspergillus parasiticus*. *Aspergillus flavus* has been investigated by contact methods and was inhibited by film incorporating sorbic acid and benzoic acid [35], natamycin [24] and carbon quantum dots [43]. The cationic peptide ε-poly-l-lysine can destroy the integrity of the plasma membrane and/or the cell walls of the fungal spores, leading to reduction of spore germination and damage to the mycelia [55]. Starch films incorporated with ε-poly-l-lysine at concentrations ranging from 1.6 to 6.5 mg/cm^2^ were more efficient against *Aspergillus parasiticus* and *Penicillium expansum* after incubation for 7 days and extended the shelf life of bread inoculated with *Aspergillus parasiticus* or *Penicillium expansum* by 1 and 3 days, respectively [56]. Interesting, the impact of the interactions between environmental conditions and these active films on reduction of aflatoxigenic fungal growth and aflatoxin production was closely dependent on relative humidity and environmental temperature. The essential oil slowly released in the vapor phase. Mateo et al. (2017) [57] proved that EVOH films with at least 0.25% (on wet base) of cinnamaldehyde effectively controlled growth of aerobic aflatoxigenic species *Aspergillus flavus* and *Aspergillus parasiticus* and aflatoxin production in maize grains (in vapor phase). The growth rate of both species was higher at 0.99 than at 0.96 a_w_ and at 37 °C than at 25 °C. Higher temperature increased molecular mobility in the film matrix, which increased the diffusion of volatile molecules [58,59]. Li et al. (2019) [60] found that chitosan films with 1.5 and 3.0 μL/cm^2^ of turmeric essential oil exhibited stronger inhibitory effect on conidial formation and inhibited the growth of *Aspergillus flavus* at 25 °C, with inactivation of the formation of green fresh spores. Production of aflatoxin was completely inhibited by steam exposure for 7 days (in vapor phase). Antifungal coating with essential oils improved the safety of nuts. Chitosan-based coating with 4% cinnamon essential oil was most effective in restricting *Aspergillus flavus* and *Penicillium citrinum* in artificially inoculated peanut kernels to 9.8% and 13.4%, respectively, after 14 days of storage at 25 °C [61], while coating solution loaded with thymol significantly inhibited the growth of mold and yeast in chestnuts stored at 0 °C (4.17 log CFU/g on day 180) and maintained the lowest decay rate (5.33%) [62].

**Table 1:** Investigations on the development of active antifungal packaging during the last five years.

Classification	Antifungal Agents	Polymer Materials	Methods of Preparation	Types of Packaging	Packaged Foods/In Vitro Antimicrobial Test	Observations	References
**Organic acids and acid salts**	Sorbic acid	PP	Extrusion molding	Film	*Aspergillus niger* ATCC 9029 (in vitro contact test) *Escherichia coli* ATCC 25922 and *Staphylococcus aureus* ATCC 29523 (in vitro contact test)	Films with 2, 4 and 6% (w/w) sorbic acid significantly inhibited the growth of *Aspergillus niger* with inhibition zones of 3.20 ± 0.40, 4.08 ± 0.60 and 6.70 ± 0.48 cm^2^, respectively, after 96 h of incubation. Antimicrobial effect of active films on Gram-negative bacteria (*Escherichia coli*) was more than that on Gram-positive bacteria (*Staphylococcus aureus*) with a decrease of 0.4 and 0.17 log cycles, respectively.	[34]
	Sorbic acid or benzoic acid	Argentine anchovy protein	Solvent casting	Film	*Aspergillus flavus* CCT 1217 and *Rhizopus oryzae* CCT 7560 (in vitro contact test)	After 24 h of incubation, *Rhizopus oryzae* was more sensitive to films with 0.50%, 0.75% and 1.50% of sorbic acid or benzoic acid than *Aspergillus flavus*. After 48 h of incubation, films with 1.50% of sorbic acid or benzoic acid showed inhibitory behavior against *Aspergillus flavus* of 8.22 and 5.55 mm, respectively, but did not affect *Rhizopus oryzae*.	[35]
	Sodium benzoate and/or potassium sorbate	PBAT/TPS blends	Blown extrusion	Film	Fresh rice noodles (stored at 25 °C for 8 days) (in vivo contact test) *Penicillium* sp. and *Aspergillus niger* (in vitro contact test)	Noodles packed with films with 3% sodium benzoate showed mold growth on day 8, while other films with sodium benzoate and/or potassium sorbate effectively inhibited mold growth during storage up to 8 days at ambient conditions and 85% RH. In vitro test, films with 3% and 6% sodium benzoate and/or potassium sorbate inhibited spore formation of *Aspergillus niger* and reduced mycelial expansion of *Penicillium* sp. During aging of 3 days, growth of both fungi covered the control film media.	[38]
**Essential oils and their constituent**	Oregano, carvacrol, cinnamon bark or cinnamaldehyde	EVOH	Solvent casting	Film	Maize grains (*Zea mays*) were inoculated with *Aspergillus flavus* and *Aspergillus parasiticus* (stored at 25 °C and 37 °C for 12 days) (in vivo vapor phase test)	The order of efficacy of films with essential oils to control growth of *Aspergillus flavus* and *Aspergillus parasiticus* and aflatoxin production in maize grains was EVOH films with cinnamaldehyde > carvacrol or oregano > cinnamon bark. The effective doses at 50% growth inhibition for films with cinnamaldehyde and cinnamon bark against *Aspergillus flavus* ranged 0.125 and 2.475–3.500 mg/plate and against *Aspergillus parasiticus* at 0.121–0.133 and 2.275–3.625 mg/plate, respectively. The effective doses at 90% growth inhibition for films with cinnamaldehyde were 0.22–0.23 mg/plate for both species.	[57]
	Oregano essential oil	Cellulose acetate	Solvent casting	Film	Hamburger buns (stored at room temperature for 30 days) (in vivo contact test)	Films with 0.5% oregano essential oil increased the shelf life of hamburger buns from 12 to 27 days by delaying the growth of filamentous fungi, while films containing 1.5%, 2.5% and 3.5% essential oil delayed the growth of filamentous fungi in hamburger buns until the 29th day.	[45]
	Eugenol and citral essential oils	Corn porous starch	Osmosis and diffusion	Microcapsules	Bread slices (stored at 25 °C for 17 days) (in vivo vapor phase test) *Penicillium roqueforti* ATCC 10110 and *Aspergillus niger* ATCC 16404 (in vitro vapor phase test)	Breads and sachets containing combinations of eugenol and citral essential oils in microcapsules placed in PP or HDPE, and LDPE packages did not show colony spots until day 14 and 16, respectively, while bread without sachets (control) presented slight mold spots on day 6 in vapor phase. In vitro test, sachets containing a combination of 0.5 g eugenol and citral essential oils significantly inhibited growth of *Penicillium roqueforti* more than *Aspergillus niger* in the gas phase after incubation for 5 days.	[25]
	Turmeric essential oil	Chitosan	Solvent casting	Film	*Aspergillus flavus* CGMCC 3.4410 (in vitro vapor phase test)	Pure films exhibited a weaker inhibitory effect on the growth and conidial formation of *Aspergillus flavus*, with inhibition growth rate in the range of 10.61 ± 2.62% to 15.57 ± 0.62% within 7 days. Films with 1.5 and 3.0 μL/cm^2^ of essential oil showed increase in conidial inhibition rates to 54.49 ± 6.35% and 58.48 ± 5.02%, respectively, and inhibition rate of *Aspergillus flavus* growth was 26.13 ± 1.87% and 28.83 ± 0.79% at 7 days, while aflatoxin production was completely inhibited by steam exposure for 7 days.	[60]
	Savory or oregano essential oil	Chia mucilage	Solvent casting	Edible film	*Aspergillus flavus* CECT 2687, *Aspergillus puulauensis* 1AO5, *Penicillium commune* 301, *Penicillium crustosum* 1AO1 and *Penicillium verrucosum* CECT 2906 isolated from pressed sheep cheese (in vitro contact test)	Films with 0.1% v/v oregano or savory essential oil did not show activity against any of the mold strains tested, while 1.0 and 1.5% v/v oregano or savory essential oil increased the diameter and percentage growth inhibition of the tested mold strains (38.01–77.66%) after incubation for 3 days. Overall, films with oregano essential oil were more active than films with savory essential oil in all cases, except in *Aspergillus puulauensis* at 1.5% v/v, which was less active than savory films.	[63]
	Thymol or R-(−)-carvone	PLA	Cast extrusion	Film	*Colletotrichum gloeosporioides* isolated from avocado and citrus (in vitro vapor phase test)	At 12 °C, films incorporated with 15% R-(−)-carvone and 20% thymol were the most effective against mycelial growth of *Colletotrichum gloeosporioides* isolated from avocado and citrus, respectively. At 25 °C, film with 20% thymol had the greatest antifungal activity against both anthracnose isolates and was significantly more effective against the avocado isolate.	[50]
	Thymol nano-emulsions	Quinoa protein/chitosan	Solvent casting	Edible film and coating	Cherry tomatoes inoculated with *Botrytis cinerea* (stored at 20 °C for 7 days) (in vivo contact test)	Tomatoes without coating and those coated with neat coating showed yeast and mold counts approximately 6.28–6.45 log CFU/cm^2^, while coating with 10% dilution of thymol nano-emulsion decreased yeast and mold to 4.7 log CFU/cm^2^ after 7 days at 20 °C.	[51]
	Thyme, cinnamon or lemongrass essential oil	Chitosan	Solvent casting	Film and coating	Peanut kernels were non-inoculated or inoculated with *Aspergillus flavus* TISTR 3041 and *Penicillium citrinum* TISTR 3437 (stored at 5 °C and 28 °C for 24 days) (in vivo contact test)	Films with 4% cinnamon essential oil showed no evidence of fungal infection at end of storage period (24 days), while control films showed around 49% of fungal infection in peanuts at 28 °C and 5 °C for 24 days. There was a gradual increase in fungal contamination of peanut kernels during storage at 28 °C. After 24 days, fungal infection was found to be 28.5% and 19.3% for films with 4% thyme and lemongrass, respectively. Similar trends were observed for different treatments of peanut kernels stored at 5 °C. In comparison to all treatments, film with 4% cinnamon essential oil coating most effectively restricted *Aspergillus flavus* and *Penicillium citrinum* contamination to 9.8% and 13.4%, respectively, in artificially inoculated peanut kernels at 28 °C for 14 days of storage.	[61]
	Thymol	PLA/PBSA blends	Blown extrusion	Film	Bread (stored at 25 °C for 14 days) (in vivo contact test) *Aspergillus* spp. and *Penicillium* spp. (in vitro vapor phase test)	Film with 3 and 6 wt% thymol delayed visible growth of yeast and mold in packed bread by 7 and 9 days, respectively, compared to 6 and 3 days in neat PLA and commercial BOPP films, respectively. Yeast and mold counts increased throughout the storage period and were 7.13, 5.83, 5.38 and <1.00 log CFU/g for BOPP, neat PLA and PLA/PBSA blends film containing 3 and 6 wt% thymol, respectively, after storage for 9 days. In vitro test, film with 3 and 6 wt% thymol showed much lower growth reduction of *Aspergillus* spp. (15.15–23.16% and 61.7–80.45%) and *Penicillium* spp. (11.49–26.21% and 55.9–74.1%), respectively, over 7 days of storage at 25 °C.	[46]
	Thymol	Chitosan nanoparticles	Coating solution	Coating	Chestnuts (stored at 0 °C for 180 days) (in vivo contact test)	Coating loaded with thymol inhibited the growth of mold and yeast in chestnuts (4.17 log CFU/g on day 180), with the lowest decay rate (5.33%) compared with uncoated control chestnuts and those coated with chitosan nanoparticles alone.	[62]
	Thyme essential oil	P(3HB-co-4HB)	Solvent casting	Film	Fresh bread pieces (stored at 25–28 °C for 15 days) (in vivo contact test) Total fresh bread molds (in vitro contact test)	Film with 30% thyme oil extended shelf life to more than 5 days (<1.00 log CFU/g) with few visible mold colonies on bread on the 10th day compared to 1–4 days (7.89 log CFU/g) for neat film. In vitro test, films with thyme oil did not show any clear inhibition zone after incubation at 25 °C for 5 days, but differences in fungal density visibly increased with decreased concentration of thyme oil loaded within films (control 0% > 10% > 20% > 30%).	[47]
	Carvacrol with and without microencapsulated by *β*-cyclodextrin	Sodium alginate	Solvent casting	Film	White mushrooms (*Agaricus bisporus*) (storage at 4 °C for 12 days) (in vivo vapor phase test) *Trichoderma* sp. isolated from postharvest white mushrooms stored at 4 °C (in vitro contact test)	At 12 days, mushrooms treated with microencapsulated carvacrol films had the lowest weight loss, exhibited excellent freshness in terms of browning index, percent open caps and overall acceptability and had lower microbial count than mushrooms packaged in films with carvacrol, pure films and those without packaging. In vitro, films with 15, 30 and 60 g/L microencapsulated carvacrol were efficient against *Trichoderma* sp. after culturing at 25 °C for 48 h, with inhibition diameters of 7.16, 9.83 and 7.34 mm, respectively. Films with 30 g/L of microencapsulated carvacrol showed better antifungal activity against *Trichoderma* sp. than the other concentrations.	[53]
	Carvacrol	Polyvinyl alcohol/corn starch blends	Solvent casting	Film and coating	Golden delicious apples were inoculated with *Botrytis cinerea* CECT-20973 and *Penicillium expansum* CECT-20906 (stored at 25 °C for 14 days) (in vivo contact test)	After incubation at 20 °C for 12 days, coatings with carvacrol decreased disease incidence level by up to 27% and by around 40% for infected fruit for *Botrytis cinerea* and *Penicillium expansum*, respectively. In the same period, damage severity was reduced by around 30%, and by up to 33%, with respect to the control samples when using carvacrol-loaded coatings for *Botrytis cinerea* and *Penicillium expansum*, respectively.	[52]
	Carvacrol nano-emulsions	Corn starch/polyvinyl alcohol blends	Solvent casting	Film	*Trichoderma* sp. Isolated from postharvest mushroom (*Agaricus bisporus*) stored at 4 °C (in vitro contact test)	Films with 15%, 20% and 25% carvacrol nano-emulsions showed inhibition against *Trichoderma* sp. with inhibition zone diameters of 20, 35 and 47 mm, respectively, after culturing at 28 °C for 48 h. However, pure films and films with 5% and 10% carvacrol nano-emulsions had no antifungal activity. When cultured for 72 h, inhibition zones were slightly narrower and spore germination was observed. Inhibition zone diameters of films with 15%, 20% and 25% carvacrol nano-emulsions were 13, 27 and 37 mm, respectively.	[54]
	Carvacrol	PLA/PBAT blends	Blown extrusion	Film	White pan bread slices and butter cake (stored at 25 °C for 8 days) (in vivo contact test) *Penicillium* sp., *Aspergillus niger* and *Rhizopus* sp. (in vitro contact test and vapor phase test)	Bread and butter cake packed in commercial PP and higher PLA control films exhibited fungal growth on day 4 of storage, while higher PBAT films with 2% and 5% carvacrol prevented mold growth and extended shelf life by 2–4 days. In vitro, blend films with 2% and 5% carvacrol had antifungal effects in both contact and release into the vapor phase by delayed mycelial darkening or sporulation of *Penicillium* sp. and *Rhizopus* sp., particularly on day 3 of incubation at 25 °C. However, results indicated unclear inhibitory effects of carvacrol-containing films on growth of *Aspergillus niger*.	[26]
	Cinnamaldehyde, eugenol or thymol nano-emulsions	Pullulan	Solvent casting	Film	*Alternaria* spp. ATCC 20084, *Aspergillus niger* ATCC 16888 and *Rhizopus stolonifer* ATCC 62276 (in vitro contact test)	Films with solid lipid nanoparticles showed higher antimicrobial activity than liquid lipid nanodroplet emulsions containing 1% and 2% of each essential oil. Films loaded with 1% cinnamaldehyde in the form of solid lipid nanoparticles showed the highest inhibition zones of 20.3, 15.9 and 18.2 mm against *Alternaria* spp., *Aspergillus niger* and *Rhizopus stolonifer*, respectively, after incubation for 4 days at room temperature.	[64]
	Cinnamaldehyde	Pullulan	Solvent casting	Film	Strawberries (stored at 3 °C for 10 days and 12 °C for 6 days) (in vivo contact test)	Films with solid lipid nanoparticles containing 1% w/w cinnamaldehyde presented significantly lower yeast and mold counts (2 log CFU/g) and total plate counts (1.5 log CFU/mL) in treated strawberries stored at 3 °C by day 10 compared to pure films. At 12 °C, significant difference between pure pullulan control films and treatment was observed for yeast and mold counts. On day 2 and day 4, reductions of 0.7 log CFU/g and 0.5 log CFU/g were observed, respectively.	[65]
	*Trans*-cinnamaldehyde	PLA/PBAT blends	Cast extrusion	Film	White pan bread slices (stored at 30 °C for 21 days) (in vivo contact test) *Penicillium* sp., *Aspergillus niger* and *Rhizopus* sp. (in vitro contact test and vapor phase test)	Higher PLA or PBAT blend films with *trans*-cinnamaldehyde (2%, 5% and 10%) effectively inhibited microbial growth of bacteria and fungi for more than 21 days at 30 °C, giving non-detected total viable counts and yeast and mold count in packed bread, except for high PLA blend films with 2% *trans*-cinnamaldehyde that showed increased total viable counts during the first 5 days (3.48 to 3.66 log CFU/g), followed by a decline. In vitro disc diffusion method, higher PLA or PBAT blend films with *trans*-cinnamaldehyde (2% and 5%) showed high antifungal efficacy against *Penicillium* sp. followed by *Aspergillus niger*. Higher PBAT blend films containing 10% *trans*-cinnamaldehyde slightly inhibited *Rhizopus* sp. For vapor phase method, only higher PLA or PBAT blend films with 10% *trans*-cinnamaldehyde effectively inhibited *Penicillium* sp. after incubation at 25 °C for 3 days.	[9]
**Natural extracts**	Garlic extract and bread aroma (containing 2-acetyl-1-pyrroline) blends	PE, EVOH or zein	Film coating	Coated PE film	Bread slices were inoculated with *Penicillium expansum* (stored at room temperature for 30 days) (in vivo contact test) *Penicillium expansum* (in vitro vapor phase test)	Film coated with zein, containing 0.5% garlic extract and 0.5% bread aroma, maintained bread free of mold infection for 30 days. Film coated with EVOH or PE, containing 0.5% garlic extract and 0.5% bread aroma, showed mold growth at day 9 and 12, respectively. In vitro, films coated with PE, EVOH or zein containing 0.5% garlic extract and 0.5% bread aroma completely inhibited growth of *Penicillium expansum*.	[48]
	Coffee parchment waste extracts	Gellan gum	Solvent casting	Film	*Fusarium verticillioides, Fusarium* sp. and *Colletotrichum gloeosporioides* (in vitro contact test)	Neat films did not show growth inhibition against *Fusarium* sp. and *Colletotrichum gloeosporioides* but growth suppression against *Fusarium verticillioides*. Film with coffee parchment waste extract (4 and 8 mg/cm^2^) showed growth inhibition against *Fusarium verticillioides*, *Fusarium* sp. and *Colletotrichum gloeosporioides* after incubation for 5 days at 30 °C, especially at the highest coffee parchment waste extract concentration (8 mg/cm^2^).	[49]
**Bacteriocins**	Natamycin	PE	Surface modification by spraying	Coated PE film	*Aspergillus niger* ANIG 001 isolated from onions (in vitro contact test)	Both coated films from natamycin/ethanol solution and natamycin/n-heptane suspension showed antifungal activity against *Aspergillus niger* with very similar inhibition zone diameters of approximately 3 cm after incubation for 7 days at 25 °C. There were no significant differences among inhibition zone diameters of coated films and all extracted coated films with tape and sonication in water.	[66]
	Natamycin	LDPE	Graft polymerization	Film	Doogh (yogurt drink) was inoculated with *Rhodotorula mucilaginosa* and *Candida parapsilosis* (stored at 25 °C for 30 days) (in vivo contact test) *Rhodotorula mucilaginosa* and *Candida parapsilosis* (in vitro contact test)	Grafted film treated at 6 min of UV irradiation was used to inhibit and control fungal contamination in Doogh samples. Grafted film caused gradual decrease in yeast growth from day 0 to day 23, with a logarithmic reduction of the yeast population from 4.07 log CFU/mL on day 0 to 1.94 log CFU/mL on day 23 (52.33%) compared to untreated film, which prolonged shelf life to 23 days during storage at 25 °C. In vitro highest diameter against *Rhodotorula mucilaginosa* and *Candida parapsilosis* was related to grafted film treated at 6 min of UV (10.62 and 8.06 mm, respectively), while lowest halo diameter was obtained for natamycin grafted film treated at 2 min of UV (7.15 and 3.79 mm, respectively).	[15]
	Natamycin	Zein or alginate	Solvent casting	Film	Kashar cheese was inoculated with *Penicillium camamberti* and *Aspergillus niger* (stored at refrigerator temperature for 45 days) (in vivo contact test) *Penicillium camamberti* was isolated from camembert cheese and *Aspergillus niger* (in vitro contact test)	Zein film, with at least 2000 ppm of natamycin, provided a constant reduction in *Aspergillus niger* (1.43 log cfu/g), while ≥1000 ppm of natamycin was adequate to inhibit the growth of *Penicillium camamberti* in kashar cheese slices for 45 days (1.19 log cfu/g). Alginate films with 2000 ppm natamycin showed antifungal activity against *Aspergillus niger* in cheese slices. The number of *Penicillium camamberti* in cheeses coated with alginate films containing 200 and 500 ppm natamycin increased during 45 days of storage. In vitro test, the maximum inhibition zone diameters were obtained for zein films with 4000 ppm natamycin at 2.20 and 3.37 cm, while alginate films with 4000 ppm natamycin brought about 4.40 and 4.97 cm for *Aspergillus niger* and *Penicillium camamberti*, respectively.	[39]
	Natamycin	Carboxymethyl cellulose	Coating solution	Coating	High moisture mozzarella cheese (stored at 7 °C for 8 days) (in vivo contact test) *Aspergillus flavus*, *Aspergillus fumigatus*, *Aspergillus niger*, *Penicillium citrinum* and *Candida albicans* (in vitro contact test)	Coating with natamycin at 0.05% and 0.5% represented a 0.6 and 0.9 log cycle reduction in yeast–mold populations, respectively. Limit was achieved on the 8th day of storage. In vitro, the strongest inhibiting performance of natamycin-carboxymethyl cellulose coating solution was reported for *Aspergillus flavus* at 0.5% w/v natamycin, while slightly weaker antagonistic activity was found in *Aspergillus niger* at coating solution with 0.05% w/v natamycin.	[24]
**Cationic peptide**	ɛ-Poly-l-lysine	Corn starch	Solvent casting	Film	Bread slices were inoculated with *Aspergillus parasiticus* and *Penicillium expansum* (stored at room temperature for 7 days) (in vivo contact test) *Aspergillus parasiticus* CECT 2681 and *Penicillium expansum* CECT 2278 (in vitro contact test)	Shelf life of bread inoculated with *Aspergillus parasiticus* was increased by 1 day for films with 1.6–6.5 mg ɛ-poly-l-lysine/cm^2^, while shelf life of bread tainted with *Penicillium expansum* was increased by 3 days with 6.5 mg ε-poly-l-lysine/cm^2^. Aflatoxins production was greatly inhibited by starch films containing ɛ-poly-l-lysine (93–100%). In vitro, films with concentrations of ɛ-poly-l-lysine ranging from 1.6 to 6.5 mg/cm^2^ were more efficient against fungal growth inhibition in solid medium for 7 days at 30 °C.	[56]
**Cationic polymer**	Polyhexamethyleneguanidine (PHMG) derivatives with organic anions: sulfanilic acid salt, stearate and granular polyethylene wax	PLA, PHB or PCL	Extrusion	Flat film	*Aspergillus niger* ATCC 16404, *Penicillium chrysogenum* ATCC 10106 and *Candida albicans* ATCC10231 (in vitro contact test)	All PHMG derivatives introduced into each film (at 0.2%, 0.6% and 1.0% PHMG) inhibited germination of *Aspergillus niger* and *Penicillium chrysogenum* incubated for 48 h at 26 °C, but reduction of live cells for both molds incubated on films with 1.0% PHMG was less than reduction of live cells for *Candida albicans*. All derivatives at concentration of 1.0% PHMG had stronger biocidal effect on cells of *Candida albicans* incubated for 48 h at 37 °C, especially with 1.0% PHMG granular polyethylene wax.	[27]
**Antagonistic yeasts**	*Williopsis saturnus* var. *saturnus*	Whey protein concentrate	Solvent casting	Edible film	*Penicillium expansum* MRC 502097 and *Aspergillus niger* MRC 200806 (in vitro contact test)	Incorporating 7 and 9 log CFU/cm^2^ *Williopsis saturnus* into films resulted in more efficient inhibition of fungal growth on agar acidified at pH 4.5 and 5.2 and incubated at 23 °C for 5 days, with decreased viability of *Penicillium expansum* and *Aspergillus niger* by 29% and 19%, respectively.	[67]
**Quantum fillers**	Carbon quantum dots	Chitosan/gelatin blends	Solvent casting	Film and coating	Avocado fruits (stored at 25 °C for 21 days) (in vivo contact test) *Aspergillus flavus* and *Colletotrichum orbiculare* (in vitro contact test) *Listeria monocytogenes* and *Escherichia coli* (in vitro contact test)	Carbon dot blend coating effectively inhibited growth of mold on the surface of avocados and extended shelf life by more than 14 days. In vitro, Films with 2 wt% carbon dots showed higher activity against *Aspergillus flavus* and *Colletotrichum orbiculare* with inhibition zones of 35 ± 5 mm and 36 ± 6 mm, respectively. Films exhibited high antibacterial activity against pathogenic bacteria, showing 100% destruction of *Listeria monocytogenes* and *Escherichia coli*.	[43]
	Sulfur quantum dots, sulfur nanoparticles or elemental sulfur	Alginate	Solvent casting	Film	Bread slices (stored at room temperature for 14 days) (in vivo contact test) *Aspergillus niger* and *Penicillium chrysogenum* (in vitro contact test) *Listeria monocytogenes* and *Escherichia coli* (in vitro contact test)	Films with 3 wt% sulfur quantum dots showed excellent inhibition of mold growth of bread wrapped for 14 days, unlike films with 3 wt% elemental sulfur or 3 wt% sulfur nanoparticles. In vitro film with 3 wt% sulfur quantum dots exhibited strong fungicidal activity against both *Aspergillus niger* and *Penicillium chrysogenum* strains, showing inhibition regions of 14 mm and 18 mm, respectively. Film with 3 wt% sulfur quantum dots or elemental sulfur showed bactericidal effect with a 2 log CFU/mL reduction of *Escherichia coli*, while film with 3 wt% sulfur nanoparticles showed a 3 log CFU/mL growth reduction. Film with 3 wt% sulfur quantum dots or sulfur nanoparticles exhibited bactericidal effect against *Listeria monocytogenes* but not for elemental sulfur film.	[44]
**Fatty acid**	Lauric acid	LDPE	Extrusion	Film	Red and yellow tree tomatoes were inoculated with *Colletotrichum tamarilloi* (stored at 24 °C for 21 days) (in vivo contact test) *Colletotrichum tamarilloi* (in vitro contact test)	Films with 3.5% w/w lauric acid obtained the best results with no presence of disease symptoms as growth of mycelia in the peduncle or skin breakage in the tree tomato after inoculation with *Colletotrichum tamarilloi* for about two weeks for the yellow variety and one week for the red variety. In vitro fungus had higher growth on the edges of the control film and film with 1.5% w/w lauric acid compared to films with concentration of 2.5% and 3.5% w/w of lauric acid.	[10]
**Metals**	ZnO nanoparticles and chitin nanoparticles blends	Bovine gelatin, gelatin nanocomposite, gelatin emulsion, two layers of gelatin nanocomposite and gelatin emulsion or polyethylene (PE)	Solvent casting	Film	Sponge cake (stored at 25 °C for 28 days) (in vivo contact test)	Comparison of fungal growth on cakes packed in pure PE film (control) showed more fungal growth on cakes than cakes in gelatin emulsion films with nanoparticles and two layers of gelatin nanocomposite and gelatin emulsion films with nanoparticles after 7, 14, 21 and 28 days of storage.	[68]
**Blends**	Potassium sorbate or grapefruit seed extract	Corn starch/chitosan/nano clay blends	Solvent casting	Film	Bread (stored at 25 °C for 20 days) (in vivo contact test) *Aspergillus niger* MTCC 1785 (in vitro contact test)	Bread sample packed in LDPE control films and film with grapefruit seed extract exhibited fungal growth on day 6 and up to 20 days at 25 °C and 59% RH, respectively. In vitro after 72 h of incubation at 37 °C, film showed a small inhibition zone of 13.47 ± 0.79 mm against *Aspergillus niger*. Antifungal activity of films with grapefruit seed extract was higher than potassium sorbate with highest zone of inhibition 25.59 ± 0.64 mm.	[69]

## 3. Antibacterial Food Packaging

### 3.1. Spoilage and Pathogenic Bacteria in Food Products

Bacteria can utilize food nutrients, obtain energy and grow under different acidity levels (pH), a_w_, temperature and the presence or absence of oxygen. Based on Gram-stain, chemical and physical properties of their cell wall structure, bacteria are classified into two broad categories as Gram-positive and Gram-negative. Bacteria are also classified according to their temperature preferences into rough categories: psychrophiles (growth range of −5 to 20 °C), psychrotrophic or psychrotolerant (growth range of −5 to 35 °C), mesophiles (growth range of 20 to 45 °C) and thermophiles (growth range of 45 to 70 °C) [20,70,71]. Many different types of bacteria can contaminate and grow in several kinds of foods, causing deterioration, spoilage and food poisoning pathogens. Bacteria of concern for food safety and food quality can be classified into two categories: food spoilage bacteria and food pathogens [72,73]. Modified atmosphere packaging (MAP) and vacuum packaging are the most popular food preservation and packaging techniques that modify ambient gas atmosphere or reduce O_2_ concentration to control microbial growth. This extends the product shelf life compared with traditional heat sealing (atmosphere condition) [74,75]. MAP and vacuum packaging accompanied by chilled storage are popular preservation methods to store fish and meat in markets [76,77]. However, the bacterial community is very diverse and some strains can grow, albeit more slowly, under oxygen-limiting conditions [78].

### 3.2. Applications of Active Packaging to Control Spoilage and Pathogenic Bacteria

Antibacterial packaging technologies are designed to kill, inhibit or retard the growth of spoilage and pathogenic bacteria that may be contaminated on the surface or within food products. Antibacterial packaging can be achieved by embedding or coating antibacterial agents in or on packaging materials to enhance food safety and quality, extend shelf life and decrease the risk of foodborne pathogens. An overview of the latest antibacterial packaging materials used in food products against both Gram-positive and Gram-negative bacteria is presented in Table 2.

#### 3.2.1. Psychrotrophic Bacteria

*Listeria monocytogenes* is a psychrotrophic Gram-positive pathogen with a growth range of 0 to 45 °C causing listeriosis infection [79], while *Staphylococcus aureus* is mostly used in tests of antimicrobial packaging. Bacteriocins are mostly incorporated in polymers to control *listeria* and *staphylococcus* growth, as they are more active against Gram-positive than Gram-negative bacteria. Aymerich et al. (2022) [80] reported that antimicrobial film based on polyvinyl alcohol with enterocin A exerted a strong antilisterial activity in vitro and in dry-cured ham stored at 8 °C, while Woraprayote et al. (2018) [81] reported that Bacteriocin 7293 diffusion coated into PLA/sawdust particle blend film was more active against Gram-positive bacteria (*Listeria monocytogenes* and *Staphylococcus aureus*) than Gram-negative bacteria (e.g., *Aeromonas hydrophila* B1, *Escherichia coli*, *Pseudomonas aeruginosa* and *Salmonella* Typhimurium) in vitro and in raw pangasius fish fillet stored at 4 °C. Similar results were found in PLA film containing sophorolipid [82]. PLA film activated with lysozyme by cold plasma showed a strong antimicrobial effect against *Listeria monocytogenes* both in vitro and in rice-milk-based smoothie stored at 10 °C [83], while PLA film containing ferulic or cinnamic acids did not show a significant antimicrobial action against *Listeria monocytogenes* due to their limited release into the aqueous culture media in vitro test. This indicated that the release of active compounds from the PLA matrix requires its plasticization, which does not occur when in contact with the culture media [84], and also the hydrophobic structure and low water affinity of the matrices may limit water absorption [85,86] that inhibits the effective release of active compounds.

#### 3.2.2. Mesophilic Bacteria

*Escherichia coli* is a Gram-negative and mesophilic bacterium with wide-ranging growth at 7 to 50 °C. *Escherichia coli* has pathogenic strains causing diverse intestinal and extraintestinal infections in humans and animals, while nonpathogenic strains are commonly found in the intestinal flora of most mammals [6]. *Staphylococcus aureus* is a Gram-positive, facultative anaerobic typical mesophile with wide-ranging growth at 7 to 48 °C and a causative agent of staphylococcal food poisoning, nausea and vomiting [70]. Recently, many researchers have used *Staphylococcus aureus* and *Escherichia coli* as models for Gram-positive and Gram-negative bacteria in antimicrobial experiments. Chitosan film incorporated with lemongrass essential oil [87], chitosan/pullulan blend film containing carvacrol [88] and PE films containing thymol or linalool [89] was more efficient against Gram-positive *Staphylococcus aureus* than Gram-negative *Escherichia coli* or *Escherichia coli* O157:H7 during in vitro testing. This occurred because Gram-positive bacteria do not have an outer membrane and are surrounded by a thick peptidoglycan wall that is not dense enough to resist small antimicrobial molecules, allowing easy penetration to the cell membrane. Jo et al. (2018) [90] reported that LDPE films incorporated with silver nanoparticles were more effective on *Staphylococcus aureus* than *Escherichia coli* due to the presence of a thick peptidoglycan layer in Gram-positive bacteria that attracts Ag^+^ ions, thus resulting in the death of the bacteria, while PP films incorporated with silver nanoparticles were equally effective on both strains. By contrast, Kim et al. (2020) [91] found that sulfur nanoparticles capped with chitosan exhibited stronger antimicrobial activity against *Escherichia coli* (Gram-negative) than *Staphylococcus aureus* (Gram-positive). Cinnamaldehyde-loaded corn starch/PBAT/PLA blend film most effectively maintained the quality of soy-protein-based meat analogs by inhibiting the growth of *Escherichia coli* and *Staphylococcus aureus* during storage at 4 °C [92]. Similarly, antimicrobial peptide ε-polylysine incorporated into levan/pullulan/chitosan edible films [93] and starch/PBAT blend film [94] gave a broad spectrum of antibacterial activity against both Gram-positive and Gram-negative pathogens due to the electrostatic attraction between ε-polylysine and the cell wall structure of bacteria, leading to leakage of intracellular components. This facilitated ε-polylysine to enter the cytoplasm, leading to abnormality in gene expressions, disturbance of oxidative stress and even cell death. Gao et al. (2022) [94] found that starch/PBAT blend film incorporated with nisin showed lower antimicrobial activity against *Escherichia coli* because the outer membrane of Gram-negative bacteria was composed of lipopolysaccharides and glycerol phospholipids that created an efficient barrier and prevented nisin from reaching the cytoplasmic membrane.

*Salmonella* is a Gram-negative mesophilic bacterium with growth in a wide range from 2 to 48 °C that causes salmonellosis in humans as well as typhoid fever, gastroenteritis and septicemia [70]. Polyvinyl alcohol/montmorillonite K10 clay nanocomposite blend films with in situ generated ginger-extract-mediated silver nanoparticles showed stronger antimicrobial activity against Gram-negative *Salmonella* Typhimurium than Gram-positive *Staphylococcus aureus* in vitro [95]. TPS/PBAT blend films coated with lauric arginate alone or combined with nisin Z dramatically decreased and eliminated *Salmonella* Typhimurium populations on inoculated raw tiger prawn slices compared to raw bigeye snapper slices at refrigerated or frozen storage [96]. Some experiments in this review on antimicrobial packaging showed lower effects on *Salmonella* Typhimurium compared to other Gram-negative or Gram-positive strains [81,97,98].

Similar to pathogens, experiments on antimicrobial active packaging against spoilage bacteria were designed as in vitro or within challenge tests. Some researchers have suggested that plant extracts embedded in certain materials slowed down the release of compounds that affect flavor and reduce the detrimental impact of these plant extracts on food flavor or taste [99,100]. Gutiérrez-García et al. (2022) [100] demonstrated that LDPE film with *Yucca baccata* butanolic extract decreased the growth of aerobic mesophile bacteria being able to extend the shelf life of ground beef, exposed to air, from 3 to 8 days at 4 °C. Guo et al. (2021) [99] demonstrated that starch film packaging containing sea buckthorn pomace extract prolonged the shelf life of beef from 25 to 35 days during super-chilled storage. The combined use of antimicrobial packaging with either modified atmosphere packaging (MAP) or vacuum packaging can enhance the effectiveness of antimicrobials as well as the shelf life of food products during cold storage (i.e., super-chilling, freezing and chilling). Some microbial groups were monitored to verify the general effectiveness of antimicrobial packaging under study, such as total viable count, total coliforms, psychrotrophic bacterial count, yeast and mold, enterobacteria and lactic acid bacteria.

Examples of antimicrobial packaging applied on foods include PBAT/TPS-blended ZnO nanocomposite films [101], refrigerated fish fillet PBAT film incorporated with oregano essential oil [102], refrigerated Pacific white shrimps packed in PLA blend film incorporated with carvacrol, citral or α-terpineol essential oils [103], frozen prawns (*Penaeus monodon*) packed in cellulose nanoparticles/polyvinyl alcohol blends incorporated with fennel seed oil [104] and slices of cooked ham packed in PP films incorporated with oregano essential oil or allium extract stored under vacuum bags at 5 °C [105].

*Yersinia enterocolitica* and *Brochothrix thermosphacta* were able to grow at temperatures as low as 0 °C, as the major pathogens or spoilage organisms in frozen ground beef [106], pork [107] and fish and seafood [108]. Contamination of foods with *Yersinia enterocolitica* and *Brochothrix thermosphacta*, especially surfaces of produce items, and their ability to survive at low temperatures during storage are a concern for both food manufacturers and consumers [108,109]. Previous studies focused on effective antimicrobial activity against spoilage and pathogenic bacteria in vitro or within challenge tests; however, scant research has addressed psychrophilic and psychrotolerant bacteria that can survive and proliferate under super-chilling or frozen conditions.

**Table 2:** Antibacterial packaging applications used in food products.

Classification	Antibacterial Agents	Polymer Materials	Methods of Preparation	Types of Packaging	Packaged Foods/In Vitro Antimicrobial Test	Observations	References
**Essential oils and their constituent**	Oregano essential oil	PBAT	Hot melt extrusion	Film	Fish fillets (stored at 7 °C for 12 days) (in vivo contact test)	Fillets packed with film containing 5%, 7.5% and 10% essential oil showed reduced and inactivated total coliforms, *staphylococcus aureus* and psychrotrophic counts that extended the shelf life of the fillets for up to 10 days.	[102]
	Oregano essential oil or allium extract	PP	Extrusion	Film	Slices of cooked ham were non-inoculated or inoculated with *Brochothrix thermosphacta* (stored at 4 °C for 60 days) (in vivo contact test)	Films with allium extract were more effective against *Brochothrix thermosphacta* than films with essential oil at 2%, 3% and 4% of allium extract, killing the bacterial population present (0.0 ± 0.0 log CFU/cm^2^) in the ham after 60 days.	[105]
	Fennel seed oil	Cellulose nanoparticles/polyvinyl alcohol blends	Solvent casting	Film	Raw prawns (*Penaeus monodon*) (stored at −20 °C for 70 days) (in vivo contact test)	Films with fennel seed oil enhanced the shelf life of prawns up to two months for both HOSO (head on shell on) prawn and PD (peeled and deveined) prawn under frozen storage, with lower microbial load than the maximum allowable limit in fish (7 log CFU/g).	[104]
	Ginger essential oil emulsion and nano-emulsions	Fish sarcoplasmic protein/chitosan blends	Solvent casting	Film	Red sea breams (*Pagrus major*) fillets (stored at 4 °C for 10 days) (in vivo contact test) Pathogenic bacteria: Gram-negative bacteria *(Escherichia coli* ATCC 25922) and Gram-positive bacteria *(Staphylococcus aureus* ATCC 6538) (in vitro contact test)	Fillets wrapped by film with 1% essential oil emulsion and nano-emulsions exhibited lower total viable counts than 6 log CFU/g for 10 days, while films with essential oil nano-emulsions (particle size 59.30 nm) had lowest total viable counts at 5 log CFU/g. In vitro, films with 1% essential oil nano-emulsions (particle size 59.30 nm) were most effective against *Escherichia coli* and *Staphylococcus aureus* with inhibition zones of 16.98 ± 0.50 and 17.70 ± 0.46 mm, respectively, after incubation at 37 °C for 24 h.	[110]
	Lemongrass essential oil	Chitosan	Solvent casting	Film	Chicken patties (stored at 4 °C for 14 days) (in vivo contact test) Pathogenic bacteria: Gram-negative bacteria (*Escherichia coli* ATCC 11229) and Gram-positive bacteria (*Staphylococcus aureus* ATCC 6538) (in vitro contact test)	Patties were stored in films with 2.5% v/w of essential oil, and the control samples showed thermotolerant coliforms count <3 log NPM/g, amount of *Clostridium* sulfite reducer was <1 log CFU/g, coagulase-positive *Staphylococcus* count <1 log CFU/g, absence in *Salmonella* count and mesophylls count <1 log CFU/g. In vitro, films with 2.5% v/w of essential oil presented more inhibition zone of *Staphylococcus aureus* than *Escherichia coli* (16.5 ± 2.1 and 11.0 ± 0.0 mm, respectively).	[87]
	Thymol or linalool	PE	Molding	Film sheet	Low-moisture mozzarella cheese (stored at 4 °C for 30 days) (in vivo contact test) Pathogenic bacteria: Gram-negative bacteria (*Escherichia coli* O157:H7), Gram-positive bacteria *(Staphylococcus aureus* ATCC 6538 and *Listeria innocua*) (in vitro contact test) Yeast: *Saccharomyces cerevisiae*	Cheese packed in films with thymol and linalool did not show *Escherichia coli* and *Staphylococcus aureus* until 30 days, while the control sample was unacceptable at 17 days of storage (17.00 and 32.05 log CFU/g, respectively). In vitro, increasing linalool or thymol at 1%, 1.5% and 2% in films increased the lag phase and reduced the proliferation of *Escherichia coli*, *Staphylococcus aureus*, *Listeria innocua* and *Saccharomyces cerevisiae* in the logarithmic phase with faster death of these microorganisms.	[89]
	Carvacrol, citral or α-terpineol essential oils	PBAT/PLA blends	Blown extrusion	Film	Pacific white shrimps (*Litopenaeus vannamei*) (stored at 4 °C for 12 days) (in vivo contact test)	Shrimps packed in films with 3% and 6% of each essential oil had significantly lower psychotropic bacteria counts than the control of up to 1.02 and 1.95 log on day 3 and day 6, respectively, while total viable count increased and exceeded the maximum limit of 7 log CFU/mL on day 9.	[103]
	Carvacrol	Chitosan/pullulan blends	Solvent casting	Film	Boer goat meat (stored at 4 °C for 15 days) (in vivo contact test) Pathogenic and spoilage bacteria: Gram-negative bacteria *(Escherichia coli* ATCC25922, *Enterobacter cloacae* CMCC(B)45301, *Pseudomonas putida* ATCC49128, *Pseudomonas fluorescens* AS1.55) and Gram-positive bacteria (*Listeria monocytogenes* ATCC19115 and *Staphylococcus aureus* ATCC6538) (in vitro contact test)	Goat meat packed with 1.50% w/v of carvacrol films remained stable for total number of colonies 3.95 log CFU/g on day 9 and still within the acceptable limit until the end of storage, while meat packed with neat film had 8.34 log CFU/g on day 9 and greater than the acceptable limit (5.0 × 10^5^ CFU/g). In vitro, films with 0.75 to 1.50% w/v of carvacrol had the best diameter of zone inhibition on *Staphylococcus aureus* (19.47 ± 1.30 mm to 33.75 ± 2.07 mm), followed by *Pseudomonas fluorescens*, *Listeria monocytogenes*, *Escherichia coli*, *Enterobacter cloacae* and *Pseudomonas putida*.	[88]
	Cinnamaldehyde or tea polyphenols	Corn starch/PBAT/PLA blends	Cast extrusion	Film	Soy-protein-based meat analogs were inoculated with Gram-negative bacteria (*Escherichia coli* ATCC 25922) and Gram-positive bacteria (*Staphylococcus aureus* ATCC 6538) (stored at 4 °C for 10 days) (in vivo contact test)	Films with cinnamaldehyde gave average reduction of *Escherichia coli* and *Staphylococcus aureus* inoculated in meat to 3.6 and 4.1 log CFU/g on day 10 at 4 °C, respectively. Differences between meat packed without film and packed with films containing tea polyphenols and neat films were not significant. Rapid increases in the bacteria population were observed from day 1 to day 7 in these three groups.	[92]
**Natural extracts**	Propolis ethanolic extract	Pullulan	Solvent casting	Film and coating	Cherry tomatoes non-inoculated or inoculated with pathogenic bacteria: Gram-positive bacteria (*Listeria monocytogenes* ATCC 7644), Gram-negative bacteria (*Salmonella* Typhimurium NIPH-NIH and *Escherichia coli* O157 ATCC 700728) or Molds: *Fusarium solani* ATCC 36031, *Penicillium chrysogenum* ATCC 10136 and *Botrytis cinerea* IOR 2110 (stored at 10 °C for 21 days) (in vivo contact test) Pathogenic bacteria: Gram-positive bacteria (*Listeria monocytogenes* ATCC 7644) and Gram-negative bacteria (*Salmonella* Typhimurium NIPH-NIH and *Escherichia coli* O157 ATCC 700728) (in vitro contact test) Molds: *Fusarium solani* ATCC 36031, *Penicillium chrysogenum* ATCC 10136, *Botrytis cinerea* IOR 2110 (in vitro contact test)	Reduction of 3.04 and 3.14 log CFU/g in the number of *Salmonella* Typhimurium occurred on tomatoes coated with 5 and 10% of extracts after 14 days, whereas the number of *Listeria monocytogenes* reduced by 3.02 and 3.28 log CFU/g on coating with 5% and 10% of extracts after 21 days, and the number of *Escherichia coli* decreased by 1.68 and 1.89 log CFU/g on coating with 5% and 10% of extracts after 7 days. Highest decrease of 1.9–2.2 log CFU/g in the number of *Penicillium chrysogenum* and *Fusarium solani* occurred on tomatoes after 7 days on coating with 5% and 10% of extracts. All three types of coatings did not inhibit the growth of *Botrytis cinerea*. In vitro, coating with 5% and 10% of extracts caused larger inhibitory zones for *Listeria monocytogenes* (12.29 ± 0.28 and 12.72 ± 1.30 mm, respectively) than *Salmonella* Typhimurium and *Escherichia coli* with no growth observed under the discs. In vitro, coating with 5% and 10% of extracts showed no inhibition of *Botrytis cinerea* growth but caused larger inhibitory zones for *Fusarium solani* (12.18 ± 1.16 and 13.14 ± 1.25 mm, respectively) than *Penicillium chrysogenum* (11.75 ± 1.03 and 13.88 ± 0.96 mm, respectively).	[98]
	Propolis ethanolic extract	PLA	Solvent casting	Film	Pathogenic bacteria: Gram-negative (*Staphylococcus aureus* ATCC 33591) and Gram-negative (*Pseudomonas aeruginosa* ATCC 9027) (in vitro contact test)	Films with 40% extracts, 10% CaCO_3_ and 15% polyethylene glycol after 24 h at 35 °C gave maximum inhibition against *Staphylococcus aureus* by 30 ± 2 mm compared to other films containing extracts, while *Pseudomonas aeruginosa* had an inhibition zone of 22 ± 1 mm.	[111]
	*Yucca baccata* butanolic extract	LDPE	Blown extrusion	Film	Ground beef (stored at 4 °C for 10 days) (in vivo contact test)	Films with 5% w/w of extracts decreased the growth of aerobic mesophilic bacteria from 3 days (6.42 ± 0.05 log CFU/g) to 8 days (6.62 ± 0.04 log CFU/g) at 4 °C. compared with control films.	[100]
	Feijoa (*Acca sellowiana* (Berg) Burret) pulp or husk extract	Brazilian pine seeds starch/citric pectin blends	Solvent casting	Film and coating	Bread slices (stored at 25 °C for 28 days) (in vivo contact test) Pathogenic bacteria: Gram-negative bacteria (*Escherichia coli* ATCC 25922, *Salmonella enterica* subsp. *enterica* serovar Typhimurium ATCC 14028 and *Shigella flexneri* ATCC 29903) (in vitro contact test)	Bread with films containing feijoa pulp and husk extract showed inhibited mold and yeast counts and increasing shelf life to 30 days of storage. In vitro, film with feijoa husk extract presented higher inhibition zones than feijoa pulp extract; *Escherichia coli* (6.70 ± 0.61 and 4.50 ± 0.50 mm), *Salmonella* (5.67 ± 0.58 and 3.33 ± 1.15 mm) and Shigella (6.33 ± 0.58 and 4.67 ± 0.58 mm), respectively, after incubation at 37 °C for 24 h.	[112]
	Crude mulberry leaf extract, chlorogenic acid or deoxynojirimycin	Pectin	Solvent casting	Film and coating	Capsicum fruit (stored at 25 °C for 12 days) (in vivo contact test) Pathogenic and spoilage bacteria: Gram-negative (*Pseudomonas aeruginosa* ATCC 27853) and Gram-positive (*Bacillus cereus* F 4810) (in vitro contact test)	Pectin blended with crude leaf extract and deoxynojirimycin-coated fruit showed increased shelf life of up to 12 days compared to 6 days in the control fruit. In vitro, diameter zone of inhibition *Pseudomonas aeruginosa* and *Bacillus cereus* found on pectin film with crude mulberry leaf extract (15.78 ± 0.06 and 18.08 ± 0.01 mm), chlorogenic acid (16.69 ± 0.21 and 16.08 ± 0.01 mm) and deoxynojirimycin (19.79 ± 0.06 and 19.36 ± 0.11 mm).	[113]
	Sea buckthorn pomace extract	Potato starch	Solvent casting	Film	Beef longissimus lumborum (LL) muscle (stored at −1.3 °C for 45 days) (in vivo contact test)	Total viable counts of films with 1%, 2% and 3% sea buckthorn pomace extract were 6.72, 5.07 and 3.92 log CFU/g, respectively, and lower than pure film that increased to 7.12 log CFU/g at the end of storage. Films with 3% of sea buckthorn pomace extract had acceptable total viable count up to 45 days. Upper limit of meat and meat products is 4.0 log CFU/g.	[99]
**Phenolic acids**	Ferulic or cinnamic acids	PLA	Melt blending and compression molding	Film	Pathogenic bacteria: Gram-positive bacteria (*Listeria innocua* CECT 910) and Gram-negative bacteria (*Escherichia coli* CECT 101) (in vitro contact test)	No antibacterial activity against *Listeria innocua* was observed for any films. Mild inhibitory effect was found against *Escherichia coli* for film (control), films with 1 or 2% ferulic acid and films with 1 or 2% cinnamic acid, but none of the film formulations caused a 2 log CFU reduction.	[84]
**Bacteriocins and cationic peptide**	Bacteriocin 7293	PLA/sawdust particle blends	Blown extrusion and diffusion coating	Coated film	Raw pangasius fish fillets were inoculated with Gram-negative bacteria (*Aeromonas hydrophila* B1, *Escherichia coli* ATCC 25922, *Pseudomonas aeruginosa* ATCC 27853 and *Salmonella* Typhimurium DMST 0562) and Gram-positive bacteria (*Listeria monocytogenes* ATCC 19115 and *Staphylococcus aureus* ATCC 25923) (stored at 4 °C for 7 days) (in vivo contact test) Pathogenic bacteria: Gram-negative bacteria (*Aeromonas hydrophila* B1, *Escherichia coli* ATCC 25922, *Pseudomonas aeruginosa* ATCC 27853 and *Salmonella* Typhimurium DMST 0562) and Gram-positive bacteria (*Listeria monocytogenes* ATCC 19115 and *Staphylococcus aureus* ATCC 25923) (in vitro contact test)	Film with Bacteriocin 7293 inhibited the viable count of all tested microorganisms inoculated on fillets by 2–5 log CFU/cm^2^ compared with the control (pure film or unpackaged). In vitro, film with Bacteriocin 7293 effectively decreased the viable counts of each indicator strain from 3.03–4.78 log CFU/cm^2^ and lower than the control after 24 h of exposure. Film with Bacteriocin 7293 was more active against Gram-positive than Gram-negative indicators.	[81]
	Lauric arginate and/or Nisin Z	TPS/PBAT (film)gelatin or pullulan (coating solution)	Blown extrusion and coating solution	Film and coated film	Bigeye snapper (*Lutjanus lutjanus*) and tiger prawn (*Penaeus monodon*) slices were inoculated with Gram-negative bacteria (*Salmonella* Typhimurium ATCC 14028 and *Vibrio parahaemolyticus* ATCC 17802) (stored at 4 °C for 28 days and −20 °C for 2 months) (in vivo contact test) Pathogenic bacteria: Gram-negative bacteria *(Escherichia coli* O157:H7 ATCC 43895, *Salmonella* Typhimurium ATCC 14028, *Salmonella enteritidis* ATCC 10118 and *Vibrio parahaemolyticus* ATCC 17802) and Gram-positive bacteria (*Listeria monocytogenes* Scott A, *Staphylococcus aureus* ATCC 12600) (in vitro contact test)	Films coated with gelatin containing lauric arginate alone or in a combination with nisin Z effectively inhibited V *Salmonella* Typhimurium and *Vibrio parahaemolyticus* on bigeye snapper and tiger prawn slices during long-term refrigerated and frozen (−20 °C) storage. In vitro, both films coated with gelatin containing lauric arginate alone or in a combination with nisin Z displayed excellent inhibition of all test strains.	[96]
	Ethyl lauroyl arginate	Chitosan/polyvinyl alcohol blends	Solvent casting	Film	Pathogenic bacteria: Gram-positive bacteria (*Listeria monocytogenes* UNIMORE 19115) and Gram-negative bacteria (*Escherichia coli* UNIMORE 40522, *Salmonella* Typhimurium UNIMORE 14028 and *Campylobacter jejuni* UNIMORE 33250) (in vitro contact test)	Films with 1% ethyl lauroyl arginate were only effective against *Campylobacter jejuni* with inhibition zone 1.8 ± 1.0 mm. Films with 5% and 10% ethyl lauroyl arginate were the most effective against all tested microorganisms, especially *Campylobacter jejuni*. In liquid medium, *Campylobacter jejuni* showed higher log reduction (7.4 ± 0.1 log CFU/mL) for films with 10% ethyl lauroyl arginate that agreed with the disk diffusion results.	[97]
	Enterocin A or ethyl lauroyl arginate	Polyvinyl alcohol	Solvent casting	Film	Sliced dry-cured hams were inoculated with Gram-positive bacteria (*Listeria monocytogenes*) (stored at 8 °C for 6 months) (in vivo contact test)	Films with enterocin A showed reduced counts of *Listeria monocytogenes* by 1.5 log units in 5 days. After 6 months, *Listeria monocytogenes* was below the quantification limit compared to the control batch (ca. 5 log CFU/g). Lower efficacy of films with ethyl lauroyl arginate in the product-specific approach was found compared to enterocin A films.	[80]
	ε-polylysine	Levan/pullulan/chitosan blends	Solvent casting	Edible films and coating	Strawberries (stored at 25 °C for 5 days) (in vivo contact test) Pathogenic bacteria: Gram-negative bacteria (*Escherichia coli* ATCC 25922) and Gram-positive bacteria (*Staphylococcus aureus* ATCC 29213) (in vitro contact test)	Coatings with ε-polylysine inhibited microbial growth on strawberry surfaces, thereby contributing to fruit firmness. In vitro, the inhibition zone diameters of all edible films with ε-polylysine against *Staphylococcus aureus* and *Escherichia coli* were in the range of 17.4–18.8 mm and 19.4–20.4 mm, respectively, after incubation at 30 °C for 24 h.	[93]
	ε-polylysine hydrochloride and/or nisin	Starch/PBAT blends	Blown extrusion	Film	Peaches (*Amygdalus persica* L. *Batsch*) (stored at 24 °C for 10 days) (in vivo contact test) Pathogenic bacteria: Gram-negative bacteria (*Escherichia coli* CVCC1387) and Gram-positive bacteria (*Staphylococcus aureus* CMCC26003) (in vitro contact test)	Peaches packaged by films with 1% ε-polylysine hydrochloride + 2% nisin had the best appearance and prolonged the shelf life until 10 days of storage. In vitro, films with 1% ε-polylysine hydrochloride + 2% nisin exhibited over 90% inhibition rates against *Staphylococcus aureus* and *Escherichia coli* after inoculation at 37 °C for 24 h.	[94]
**Enzymes**	Lysozyme	PLA	Cold plasma treatment	Coated film and pouch	Pear juice and rice-milk-based smoothie were inoculated with Gram-positive bacteria (*Listeria monocytogenes* Scott A and *Lactobacillus plantarum* 82) (stored at 4 °C for 16 days and 10 °C for 10 days) (in vivo contact test) Pathogenic bacteria: Gram-positive bacteria (*Listeria monocytogenes* Scott A, *Listeria monocytogenes* ATCC13932, *Listeria innocua* ATCC51742, *Listeria innocua* DSM2029Y, *Staphylococcus aureus* SR41 and *Enterococcus faecium* t2) (in vitro contact test) Spoilage bacteria: *Lactobacillus plantarum* 82 and *Pediococcus damnosus* 11 (in vitro)	Antimicrobial effect of lysozyme-activated pouches on *Lactobacillus plantarum* was lower compared to *Listeria monocytogenes* during 10 days of storage at 10 °C. Activated pouches showed the best antimicrobial effect on smoothie samples stored at 10 °C compared to 4 °C. In vitro, lysozyme-activated film showed good efficacy against *Enterococcus faecium*, *Listeria monocytogenes* Scott A, *Pediococcus damnosus* and *Lactobacillus plantarum*, with diameter halos ranging between 22.34 and 18.34 mm depending on the microorganism.	[83]
**Metals**	Silver (Ag) nanoparticles	LDPE	Corona air plasma treatment	Coated film and pouch	Pasteurized milk (stored at 4 °C for 16 days) (in vivo contact test)	Microbial load of milk packaged with Ag nanoparticle coated films at input powers of 210, 500 and 800 W reached the maximum limit after 5, 8 and 14 days, respectively.	[114]
	Silver (Ag) nanoparticles	LDPE or PP	Extrusion	Film	Pathogenic bacteria: Gram-negative bacteria (*Escherichia coli* ATCC 8739) and Gram-positive bacteria (*Staphylococcus aureus* ATCC 6538P) (in vitro contact test)	After 24 h of incubation at 37 °C, a substantial decrease in the number of bacteria was observed, particularly for LDPE films with 36 mg/kg of Ag nanoparticles and PP films with 30 mg/kg of Ag nanoparticles, which showed reductions above 99.9% of the original inoculum.	[90]
	Silver (Ag) nanoparticles	LDPE	Blown extrusion	Film	Olivier salad (stored at 4 °C for 22 days) (in vivo contact test)	Total bacterial count and mold reduced with time or increase in Ag nanoparticle concentration and resisted bacterial growth, while the coliform count was not affected by Ag nanoparticle concentration and decreased as a function of time. No *Escherichia coli*, *Staphylococcus aureus*, *Clostridium perfringens*, *Bacillus cereus* and *Salmonella* growth were found in the samples.	[115]
	Zinc oxide (ZnO) nanoparticles	PBAT/TPS blends	Blown extrusion	Film	Ground pork (stored at 4 °C for 12 days) (in vivo contact test)	After storage for 9 days, total viable count in meat packaged in control film exceeded the limit, while films with ZnO (1–5%) effectively retained total viable count values below 7 log cfu/g. Films with 1% and 2% ZnO had a shelf life of approximately 12 days, while 3%, 4% and 5% ZnO effectively delayed microbial growth and increased pork shelf life to more than 12 days.	[101]
**Glycolipid biosurfactant**	Sophorolipid	PLA	Solvent casting	Film	Pathogenic bacteria: Gram-positive bacteria (*Listeria monocytogenes* and *Staphylococcus aureus*) and Gram-negative bacteria (*Salmonella* spp.) isolated from poultry meat (in vitro contact test)	After incubation at 37 °C for 24 h, *Listeria monocytogenes* and *Staphylococcus aureus* were completely inhibited in films with 10% of sophorolipid, representing more than >4 log reduction and >2 log reduction, respectively. Regarding *Salmonella* spp., no bactericidal action was observed under the maximum concentration 20% of sophorolipid, although it was possible to observe a significant bacterial population reduction of 50%.	[82]
**Blends**	Silver (Ag) nanoparticles and/or ginger extract	Polyvinyl alcohol/montmorillonite K10 clay nanocomposite blends	Solvent casting	Film and pouch	Raw chicken sausage (stored at 4 °C for 4 days) (in vivo contact test) Pathogenic bacteria: Gram-negative bacteria (*Salmonella* Typhimurium MTCC 1251) and Gram-positive bacteria (*Staphylococcus aureus* MTCC 96) (in vitro contact test)	Films with ginger extract and Ag nanoparticles were highly efficient in reducing the microbial burden in samples compared to the control polyethylene pouches after 4 days of incubation at 4 °C. In vitro, films with ginger extract and Ag nanoparticles showed clear antimicrobial activity against both *Salmonella* Typhimurium and *Staphylococcus aureus*, with significant action in the Gram-negative bacterium *Salmonella* Typhimurium after incubation at 37 °C for 24 h.	[95]

## 4. Antiviral Food Packaging

### 4.1. Foodborne Virus Pathogens

Currently, interest in viruses has been increasing as the cause of foodborne outbreaks worldwide. Unlike bacteria, viruses do not affect food spoilage and are not able to grow or multiply in raw materials and processed foods during storage. Food products may become contaminated either directly or indirectly at all stages of the food chain by fecal contamination, cross-contamination during processing or by infected food handlers [116,117]. Foodborne enteric viruses are primarily transmitted by the fecal–oral route, increasingly recognized as a significant food safety concern, and have been globally associated with food and waterborne illness. Enteric viruses are robust and stable in the gastrointestinal tract (such as low pH in the stomach or high bile concentration). They also survive extremely well in the environment under various food processing and storage conditions [118]. Important enteric viruses in foodborne illness outbreaks include hepatitis A virus (HAV) and human norovirus (known as Norwalk virus; NoV) [119].

Human norovirus (NoV) is the major cause of epidemic nonbacterial outbreaks of gastroenteritis in humans worldwide, while hepatitis A virus (HAV) is the leading cause of foodborne illness [7,8]. Sánchez and Bosch (2016) [7] extensively reviewed the stability of enteric viruses in food products. Hepatitis A virus (HAV) and human norovirus (NoV) can survive on chilled food, under frozen storage, under modified atmosphere packaging (MAP) and on acidic or dried food products for long periods before deterioration of the specified food. Outbreaks due to hepatitis A virus (HAV) are most often associated with the consumption of contaminated drinking water, fruit and fruit juices, milk and milk products, frozen produce (vegetables and berries) and raw or under-cooked foods, as well as shellfish and bivalve mollusks from contaminated water [120,121]. Human norovirus (NoV) outbreaks are related to the consumption of contaminated municipal drinking water, well water, commercial ice, frozen produce (vegetables and berries), fruits and salad, as well as bivalve molluscan shellfish (such as oysters, mussels and clams) [122,123,124]. Several strategies are available to inactivate or reduce the number of enteric viruses present in food including using sanitizers or disinfectants (such as chlorine, chlorine dioxide, organic acids, peroxyacetic acid, ozone and electrolyzed water) for washing fruits and vegetables, traditional thermal processing technologies and nonthermal processing technologies (such as high-pressure processing (HPP), high-power ultrasound (HPU), high-intensity pulsed light (HIPL) or ultraviolet light (UV) disinfection) [125,126].

**Figure 2:**
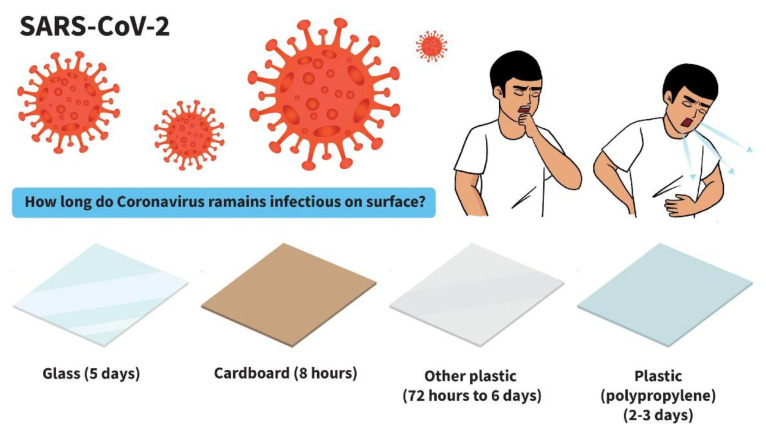
Contamination of SARS-CoV-2 viruses on packaging surface and transfer via touching the nose, mouth or eyes, which leads to disease spread more rapidly through human-to-human interactions, e.g., breathing, sneezing or coughing.

Severe acute respiratory syndrome coronavirus 2 (SARS-CoV-2), also known as coronavirus, is a respiratory virus and the leading cause of the coronavirus disease 2019 (COVID-19) pandemic. The World Health Organization (WHO) declared the coronavirus disease 2019 (COVID-19) outbreak as a pandemic health emergency on 11 March 2020. Unlike other viruses, coronavirus can contaminate and survive on surfaces, e.g., food products, food packaging materials, shipping boxes or objects such as elevator buttons, doorknobs or light switches for several days [127,128]. The long stability of SARS-CoV-2 on the surfaces of packaging materials created substantial risks during handling production until consumption. Cross-contamination hazards from infected food handlers are also one of the major routes of transmission of the virus via food [129]. Recent research has confirmed that coronavirus remains infectious on surfaces including glass (5 days), cardboard (8 h), silicon (5 days), PVC and Teflon (5 days), plastic (polypropylene) (2–3 days) and other plastics (72 h to 6 days) at room temperature [130,131]. Figure 2 presents contamination of SARS-CoV-2 viruses on packaging surface. The virus can be transmitted via touching contaminated surfaces and then touching the nose, mouth or eyes and the disease can spread rapidly through human-to-human interactions, e.g., breathing, sneezing or coughing [132]. The COVID-19 pandemic has had a severe impact on the food supply and safety, as well as food security. Han et al. (2021) [133] discussed inactivation methods and control of coronavirus in the food system by using chemical disinfectants (e.g., ethanol, sodium hypochlorite or hydrogen peroxide) to reduce the spread via contact surfaces, using heat treatment to inactivate viruses in food products, ultraviolet (UV) treatment for surface disinfection in hospital rooms and microbial inactivation in food safety applications. Unfortunately, food processing generally includes heat treatments and can lead to undesirable effects both in nutritional and sensorial attributes of fresh food products or fresh produce, while chilling or freezing treatment is not sufficient to eliminate viral transmission or reduce food viral loads [122,134]. Novel food packaging with antiviral activity is a topic of great interest both in academia and the research industry due to the growing consumer demand for safe and long-shelf-life foods.

### 4.2. Applications of Active Packaging to Control Foodborne Viruses

Antiviral packaging materials for food applications are designed to improve food safety by either preventing cross-contamination on food surfaces or inactivating target-specific foodborne viruses. Zein, gelatin, alginate, carrageenan, chitosan and poly (lactic acid) (PLA) have been successfully used as biopolymer carriers of antiviral agents. Norovirus surrogates such as feline calicivirus and murine norovirus are most widely used to study the resistance of human norovirus. Among these, murine norovirus (MNV-1) is often used as a surrogate to study the resistance of human norovirus to antimicrobial packaging because it shares a similar genome organization with human norovirus and can infect cells in culture as well as replicate in the gastrointestinal tract of its host [135]. Carrageenan-based coatings incorporating green tea extract were slightly more effective in reducing murine norovirus (MNV) than hepatitis A virus (HAV) in inoculated blueberries and raspberries [136]. Some biopolymers containing active compounds show stronger effects on reducing both murine norovirus (MNV) and hepatitis A virus (HAV) such as alginate/lipid blend films containing green tea extract or grape seed extract [137] and alginate-oleic acid-based coatings incorporating green tea extract [138]. Temperature is an important factor that influences antiviral action [139]. The infectivity of human enteric viruses in fresh berries after coating treatments was higher at room temperature compared to refrigerated storage; however, viruses usually persist better at lower than higher temperatures [138,140].

Recent developments in antiviral biodegradable food packaging and edible coating as well as their applications on food products are shown in Table 3.

Increased development of biopolymer-based food packaging with antiviral activity has occurred, but little development has addressed (i) biodegradable packaging with antiviral activity via commercial production such as blown extrusion, cast extrusion or compression molding, (ii) development of multifunctional biodegradable packaging with antiviral activity via commercial production such as mono- or multilayered films, consisting of inner layers with antifungal or antibacterial activity to extend the shelf life of food products and outer layers with antiviral activity to prevent re-contamination and (iii) application on various foods served raw including fruits and vegetables and shellfish and other seafood that can easily become contaminated by viruses anywhere in the supply chain, especially during cold storage. All knowledge gained from the large number of existing studies on antimicrobial polymers can be effectively utilized to develop multifunctional antimicrobial materials. Further investigations are required to develop new antiviral packaging materials to protect food products and packaging surfaces from SARS-CoV-2 contamination. Moreover, the antimicrobial agents and the polymeric matrices reported have wider potential applications in the biomedical and textile fields [141,142], The antimicrobial mechanism need to be clarified to achieve processability for the desirable application.

**Table 3:** Antiviral packaging applications used in food products.

Classification	Antiviral Agents	Polymer Materials	Methods of Preparation	Types of Packaging	Packaged Foods/In Vitro Antimicrobial Test	Observations	References
**Essential oils and their constituents**	Allyl isothiocyanate	Persian gum/gelatin blends	Solvent casting	Edible film and coating	Blueberries (*Vaccinium corymbosum*) were inoculated with murine norovirus (MNV-1 strain) (stored at 10 °C and 25 °C overnight incubation) (in vivo contact test)	Films with 0.1% and 0.5% allyl isothiocyanate effectively reduced MNV titers by 1.58 and 2.79 log TCID_50_/mL, respectively, after incubation at 10 °C compared with fruits coated with control film but with low antiviral activity at 25 °C.	[143]
**Natural extracts**	Green tea extract or grape seed extract	Alginate/lipid blends	Solvent casting	Edible film	Norovirus surrogates: murine norovirus (MNV-1 strain) (in vitro contact test) Enteric virus: hepatitis A virus (HAV; HM-175/18f strain) (in vitro contact test)	Films with 0.75 g of green tea extract and grape seed extract decreased MNV titers by 1.92 and 1.67 log TCID_50_/mL, respectively. HAV titers decreased by 1.92 and 1.50 when treated with films containing 0.75 g of green tea extract and grape seed extract, respectively.	[137]
	Green tea extract	Alginate/oleic acid blends	Solvent casting	Edible film and coating	Strawberries (*Fragaria x ananassa*) and raspberries (*Rubus idaeus* L.) were inoculated with murine norovirus (MNV-1 strain) and hepatitis A virus (HAV) (stored at 10 °C for 4 days and 25 °C overnight incubation) (in vivo contact test) Norovirus surrogates: murine norovirus (MNV-1 strain) (in vitro contact test) Enteric virus and hepatitis A virus (HAV; HM-175/18f strain; ATCC VR-1402) (in vitro)	MNV and HAV in fresh strawberries after coating treatments reduced by 1.5–2 log TCID_50_/mL during 4 days of storage at 10 °C, and complete inactivation of both viruses was observed after overnight storage at 25 °C. In vitro, MNV showed higher decreasing titers by 3.42 and 5.76 log TCID_50_/mL for films with green tea extract at pH 7.0 and 5.5, respectively, after overnight incubation at 37 °C. By contrast, films with green tea extract at pH 7.0 had almost no effect against HAV after incubation at 10, 25 and 37 °C.	[138]
	Green tea extract	κ-, ι -, λ-carrageenan	Solvent casting	Edible film and coating	Blueberries and raspberries were inoculated with murine norovirus (MNV-1 strain) and hepatitis A virus (HAV; HM-175/18f strain; ATCC VR-1402) (stored at 10 °C for 4 days and 25 °C overnight incubation) (in vivo contact test)	HAV titers in blueberries were reduced by 2.88, 2.92 and 1.83 log TCID_50_/mL after overnight incubation at 25 °C for κ-, ι- and λ-carrageenan coatings with green tea extract, respectively. At refrigerated temperature, coated raspberries showed higher decrease in HAV titers by 1.79, 1.75 and 1.71 after overnight incubation for κ-, ι- and λ-carrageenan coatings with green tea extract, respectively. MNV titers in coated raspberries were significantly reduced by 2.25 and 2.79 log TCID_50_/mL for ι- and λ-carrageenan coatings containing green tea extract, respectively.	[136]
	Grape seed extract	Chitosan	Solvent casting	Film	Norovirus surrogates: murine norovirus (MNV-1) (in vitro contact test) Photogenic bacteria: *Listeria innocua* ATCC 33090 and *Escherichia coli* K12 ATCC 29181 (in vitro contact test)	Films with 5%, 10% and 15% grape seed extract caused MNV reductions of 0.92, 1.89 and 2.27 log PFU/mL, respectively, after 4 h of incubation. After 24 h, the 5% and 10% grape seed extract films reduced MNV titers by 1.90 and 3.26 log PFU/mL, respectively, while the 15% grape seed extract film reduced MNV to undetectable levels. *Escherichia coli* K12 showed reductions of 2.28, 5.18 and 7.14 log CFU/mL after 24 h exposure by 5%, 10% and 15% grape seed extract films, respectively. *Listeria innocua* counts were reduced by 3.06, 6.15 and 6.91 log CFU/mL by 5%, 10% and 15% grape seed extract films, respectively.	[144]
	*Larrea nitida* extract	Agar, alginate or agar/alginate blends	Solvent casting	Edible film and coating	Blueberries were inoculated with murine norovirus (MNV-1) (stored at 10 °C for 4 days and 25 °C overnight incubation) (in vivo contact test)	Coatings with *Larrea nitida* extract reduced MNV titers by 1.37, 0.88 and 0.92 logs compared to neat coatings, after overnight incubation at 25 °C, overnight incubation at 10 °C and 4 days at 10 °C.	[140]
**Metals**	Silver (Ag) nanoparticles	Poly (3-hydroxybutyrate-co-18 mol%-3-hydroxyvalerate) (PHBV18)	Electrospinning	Fiber mats and coated films	Norovirus surrogates: murine norovirus (MNV-1 strain) and feline calicivirus (F9 strain, ATCC VR-782) (in vitro contact test)	Electrospun coating completely inactivated FCV, while MNV titers decreased by 0.86 log.	[145]

## 5. Conclusions and Future Perspectives

Copious research has been conducted on antimicrobial food packaging applications developed from different pure/blends of biopolymers by extrusion or coated film (PBAT, PLA and starch) and solvent casting (chitosan, cellulose, pullulan, pectin, gellan gum and zein). Biobased antimicrobial packaging can reduce the use of chemical preservatives in food products and lower the consumption of plastic-materials-based packaging. Essential oils and their constituents and silver nanoparticles as antimicrobial agents are now gaining extensive attention in the active packaging field to improve the antimicrobial activities of materials against spoilage and pathogens, yeasts, molds, bacteria and viruses. Antifungal packaging efficiency inhibits yeast and mold spoilage by either direct contact or indirect contact. Very low temperatures can reduce antimicrobial agent migration rates from packaging materials, resulting in lower antimicrobial performance than in vitro and challenge test storage at higher temperatures. Antimicrobial material (i.e., films, coated films, coatings and pouches) exhibits highly efficient antimicrobial activity in in vitro assays but lower activity in real food systems that depend on (i) polar or non-polar food components, (ii) interactions between antimicrobial compounds and polymer materials and (iii) interactions between environmental conditions and active films (i.e., relative humidity, oxygen and water vapor permeability and temperature) that closely relate to the migration or diffusion of active compounds in foods. Combining antibacterial packaging with food preservation methods such as modified atmosphere packaging (MAP), vacuum packaging, super-chilling, freezing and chilling enhance the effectiveness of antimicrobials while increasing the shelf life of foods of animal origin. Recent antivirus packaging material for food applications based on biodegradable and natural plant extracts using solvent casting exhibited appropriate antiviral activity to reduce the foodborne viruses murine norovirus (MNV) and hepatitis A virus (HAV) in vitro, and the challenge test was used on coated berries at refrigerated and room temperatures. Further studies on packaging development with antiviral activity via commercial production methods using multifunctional antimicrobial materials, such as mono- or multilayered films consisting of an inner layer with antifungal or antibacterial activity to extend the shelf life of food products and an outer layer with antiviral activity to evaluate package effectiveness against novel coronavirus or related pathogens, are required. All knowledge gained from the multitude of existing studies on antimicrobial polymers can be effectively utilized to develop effective packaging to protect food products from SARS-CoV-2 contamination.

## Data Availability

The data presented in this study are available on request from the corresponding author.
